# Pim-1 kinase phosphorylates RUNX family transcription factors and enhances their activity

**DOI:** 10.1186/1471-2121-7-21

**Published:** 2006-05-09

**Authors:** Teija LT Aho, Jouko Sandholm, Katriina J Peltola, Yoshiaki Ito, Päivi J Koskinen

**Affiliations:** 1 Turku Centre for Biotechnology, University of Turku/Åbo Akademi University, Tykistökatu 6 B, 20520 Turku, Finland; 2 Turku Graduate School of Biomedical Sciences, University of Turku, Kiinamyllynkatu 13, 20520 Turku, Finland; 3 Institute of Molecular and Cell Biology, 61 Biopolis Drive, Singapore 138673, Singapore

## Abstract

**Background:**

The *pim *family genes encode oncogenic serine/threonine kinases which in hematopoietic cells have been implicated in cytokine-dependent signaling as well as in lymphomagenesis, especially in cooperation with other oncogenes such as *myc*, *bcl*-2 or *Runx *family genes. The *Runx *genes encode α-subunits of heterodimeric transcription factors which regulate cell proliferation and differentiation in various tissues during development and which can become leukemogenic upon aberrant expression.

**Results:**

Here we have identified novel protein-protein interactions between the Pim-1 kinase and the RUNX family transcription factors. Using the yeast two-hybrid system, we were able to show that the C-terminal part of human RUNX3 associates with Pim-1. This result was confirmed in cell culture, where full-length murine Runx1 and Runx3 both coprecipitated and colocalized with Pim-1. Furthermore, catalytically active Pim-1 kinase was able to phosphorylate Runx1 and Runx3 proteins and enhance the transactivation activity of Runx1 in a dose-dependent fashion.

**Conclusion:**

Altogether, our results suggest that mammalian RUNX family transcription factors are novel binding partners and substrates for the Pim-1 kinase, which may be able to regulate their activities during normal hematopoiesis as well as in leukemogenesis.

## Background

The *pim*-1 proto-oncogene was first identified as a common proviral insertion site associated with murine leukemiavirus-induced lymphomagenesis, and its oncogenic activity was verified with transgenic mice overexpressing *pim*-1 in the lymphoid compartment [1]. These mice show a low incidence of spontaneous T-cell lymphomas, the development of which can be accelerated by activation of cooperating oncogenes, such as *myc *family genes, *bcl*-2 or *Runx2 *[1-3]. Two additional, functionally redundant *pim *family members have been identified with partially overlapping expression patterns. The murine *pim*-1 gene encodes 44 and 34 kD isoforms of a serine/threonine-specific kinase [4], whose expression in hematopoietic cells can be induced by a variety of cytokines, such as interleukins 2, 3, 6 and interferon-α [5-7]. We and others have shown that Pim-1 is involved in cytokine-dependent signaling via its ability to regulate activities of the NFATc [8] and c-Myb [9] transcription factors, the Epstein-Barr virus nuclear antigen-2 [10] and the SOCS family suppressors of cytokine signaling [11,12]. Pim kinases also enhance hematopoietic cell survival and participate in regulation of the cell cycle [13].

RUNX family proteins (also known as AML, PEBP2α or CBFα) [14] are DNA-binding α-subunits of heterodimeric transcription factors that are essential for both cell proliferation and differentiation during development [15]. Homozygous disruption of murine *Runx2 *results in complete lack of bone formation, *Runx1 *knockout mice are embryonally lethal due to failure of definitive hematopoiesis, and *Runx3*-deficient mice display abnormal development of gastric epithelium and dorsal root ganglion as well as defects in thymopoiesis. In addition, strict spatiotemporal expression of all *Runx *family genes is critical for normal hematopoiesis [16]. The RUNX proteins contain an evolutionary conserved region, the Runt domain, which has been named after their structural homologue in *Drosophila *[17]. This region is required for DNA-binding as well as for dimerization with the β-subunit. While three mammalian genes encode α-subunits: RUNX1 (PEBP2αB), RUNX2 (PEBP2αA) and RUNX3 (PEBP2αC), only one gene has been identified for the β-subunit (PEBP2β/CBFβ). The β-subunit can enhance DNA-binding by the Runt domain but does not contact DNA itself [15,18]. There is less sequence similarity between RUNX family members outside the Runt domain, except for the highly conserved five amino acid C-terminus (VWRPY) known to bind transcriptional repressors, but the C-terminal regions are rich in proline, threonine and serine (PTS) and contain domains involved in transcriptional activation or inhibition [19]. RUNX activity has recently been shown to be regulated by several extracellular signaling pathways resulting in post-translational modifications, such as phosphorylation, acetylation and ubiquitination. [20].

The involvement of *RUNX *genes in cancer was first discovered as chromosomal translocations associated with acute myeloid leukemia [21]. These translocations had resulted in fusion proteins lacking the C-terminal transactivation domains of RUNX1. Evidence for *Runx1 *function as a tumor suppressor gene was obtained from knock-in mice where a single *Runx1-eto *fusion allele caused a similar phenotype as observed for the *Runx1 *null mice [22,23]. Human *RUNX3 *has also been shown to act as a tumor suppressor in gastric carcinomas [24]. However, recent retroviral tagging studies have indicated that any of the three murine *Runx *genes can also operate as dominant oncogenes that can co-operate with *myc *and *pim *family genes in lymphomagenesis [3,25]. Human *RUNX *genes have also been observed to be amplified in childhood leukemias [26,27].

Here we show that the Pim-1 kinase can physically interact with RUNX family transcription factors, colocalize with them within nuclei and phosphorylate them *in vitro*. Furthermore, the transactivation ability of Runx1 is potentiated by Pim-1, suggesting a mechanism via which Pim-1 may regulate the activity of RUNX family transcription factors during hematopoiesis as well as in leukemogenesis.

## Results and discussion

### Pim-1 interacts with RUNX family proteins

To search for putative Pim-1-interacting partners, we used the yeast two-hybrid system as previously described [9,28]. A kinase-deficient K67M mutant of Pim-1 fused to the LexA DNA-binding domain was used as a bait to screen a library of cDNA clones that had been isolated from Epstein-Barr virus-transformed human lymphocytes and fused to the VP16 activation domain. Out of the approximately 6 × 10^6 ^yeast transformants tested, 220 clones were recovered that were able to activate two separate reporter genes in a strictly Pim-1-dependent fashion.

**Figure 1:**
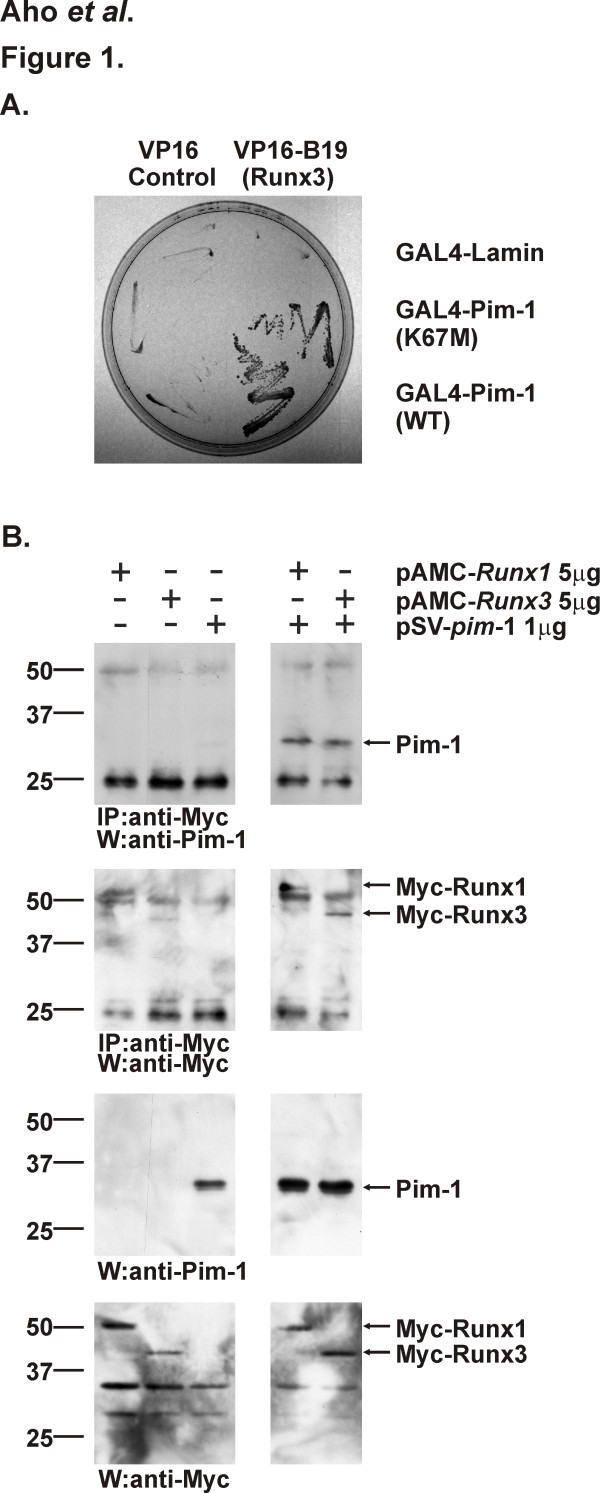
**Pim-1 interacts with RUNX family proteins**. (A) Yeast strains expressing the VP16 activation domain alone or fused with the B19 fragment of human RUNX3 were mated with strains expressing the GAL4 DNA-binding domain fused with the control protein lamin or either kinase-deficient (K67M) or wild-type (WT) Pim-1. The ability of two proteins to interact with each other was judged based on the capacity ot the corresponding diploid strains to grow on the selective medium lacking histidine. (B) COS-7 cells were transfected with pSV-*pim*-1, pAMC-*Runx1 *or pAMC-*Runx3 *plasmids as indicated in the figure. Parts of the cell lysates were subjected to immunoprecipitation with anti-Myc antibody followed by immunoblotting with anti-Pim-1 or anti-Myc antibodies. The expression of proteins in the lysates was verified by direct Western blotting with the same antibodies.

Sequence analysis revealed that one of the strongly interacting cDNA clones, designated B19, encoded amino acids 264–404 of the human RUNX3 protein, including its C-terminal transactivation and inhibition domains, but lacking the most C-terminal end (see Figure 3B). To test whether this fragment interacted also with the wild-type Pim-1 protein, a mating assay was carried out using a modified two-hybrid assay [29] with baits fused to the GAL4 DNA-binding domain. Results from this assay indicated that the VP16-B19 fusion protein interacts equally well with both the wild-type and mutant GAL4-Pim-1 fusion proteins, but not with any control proteins tested, such as lamin or the Src kinase, as judged by the ability of the diploid yeast strains to grow on selective medium lacking histidine (Figure 1A and data not shown). The bait proteins did not activate the reporter genes on their own in the absence of the B19 fragment, while all strains were able to grow on non-selective medium containing histidine (Figure 1A and data not shown). The physical interaction observed *in vivo *in yeast cells was also biochemically confirmed in a GST pull-down assay, where bacterially produced GST-B19 fusion protein was specifically able to associate with *in vitro *translated ^35^S-labeled Pim-1 protein (data not shown).

To further examine the interaction between Pim-1 and full-length RUNX transcription factors within living cells, COS-7 cells were transiently transfected with vectors expressing Pim-1 and either MYC-tagged Runx1, Runx3 or FLAG-tagged Runx1. Two days later, cells were collected and lysed, after which the cell lysates were subjected to immunoprecipitation with anti-MYC or FLAG antibodies followed by Western blotting with anti-Pim-1 antibody. This analysis revealed that Pim-1 can be coprecipitated together with both Runx1 and Runx3 full-length proteins (Figure 1B and data not shown).

### Pim-1 colocalizes with Runx1 and Runx3 proteins in nucleus

**Figure 2:**
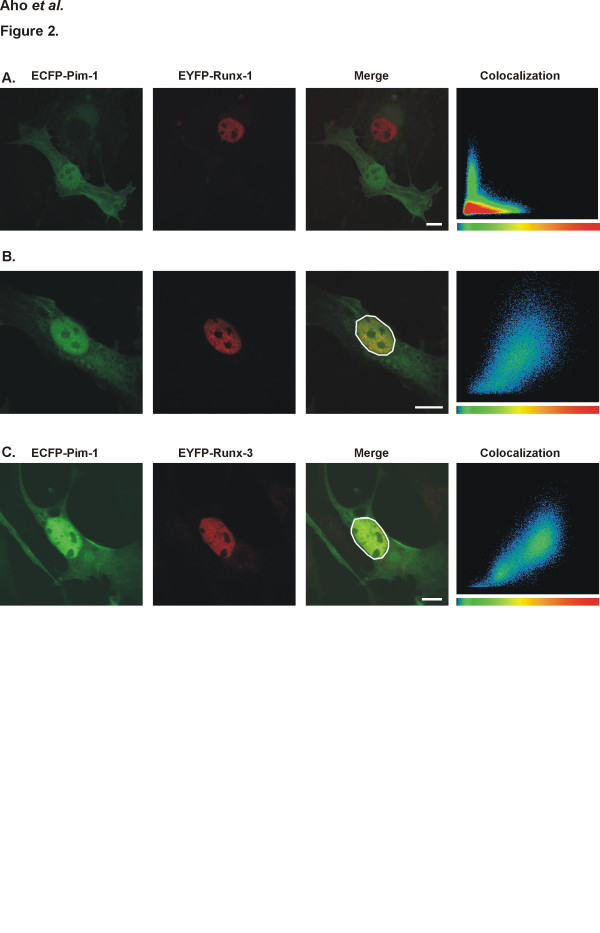
**Pim-1 colocalizes with Runx-1 and Runx-3**. The subcellular distribution of ECFP-Pim-1, EYFP-Runx1 and EYFP-Runx3 was analysed from transiently transfected COS-7 cells under confocal microscope. Shown are single- (A) or double-positive (B, C) cells expressing indicated fluorescent proteins. Colocalization of ECFP (first panel) and EYFP (second panel) fusion proteins in the circled nuclei was visualized by yellow colour in merged images (third panel) and was confirmed by scattergram plots (fourth panel), where the intensities of the CFP and YFP channels are on the X- and Y-axis, respectively. Bar represents 20 μm.

To be able to investigate the intracellular distribution and possible colocalization of Pim-1 and RUNX proteins, COS-7 cells were transiently transfected with vectors expressing Pim-1 fused to the enhanced cyan fluorescent protein (ECFP) and either Runx1 or Runx3 fused to the enhanced yellow fluorescent protein (EYFP). As expected from previous studies with untagged proteins [30-32], Pim-1 protein was found both in the nucleus and the cytoplasm of interphase cells, while Runx1 and Runx3 proteins predominantly localized to the nuclei in a granular expression pattern (Figure 2). More intriguingly, both Runx1 and Runx3 colocalized with Pim-1 in the nuclei of double-positive cells, as demonstrated by scattergram analysis of merged fluorescent images from fixed as well as from living cells (Figures 2B, 2C and data not shown). Altogether, our immunoprecipitation and imaging results indicated that Pim-1 can colocalize and physically interact with Runx family proteins within the nuclear compartment.

### Pim-1 can phosphorylate Runx1 and Runx3 proteins *in vitro*

**Figure 3:**
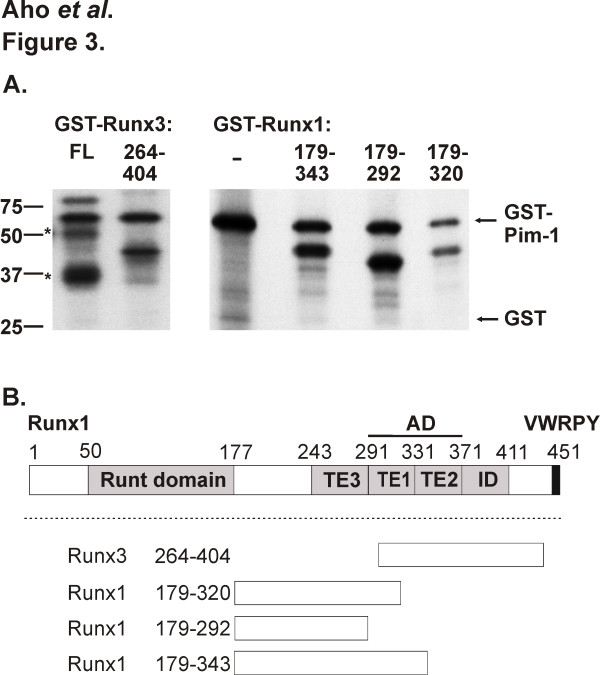
**Pim-1 phosphorylates Runx proteins *in vitro***. (A) Bacterially produced GST fusion proteins expressing either full-length (FL) or fragments of Runx1 or Runx3 were incubated with GST-Pim-1 in *in vitro *kinase assays. The phosphorylation products were separated on SDS-PAGE and visualized by autoradiography. GST alone (-) was used as a negative control. * indicates protein degradation products. (B) Schematic presentation of the functional domains of Runx1, including the Runt domain, an activation domain (AD) with two major transactivation elements (TE1 and TE2), a minor transactivation element (TE3), an inhibitory domain (ID) and the C-terminal VWRPY sequence. Shown are also the fragments phosphorylated by Pim-1 in Runx1 or Runx3, which lacks the sequences corresponding to TE3 of Runx1.

To find out whether human or murine RUNX proteins act as substrates for the Pim-1 kinase, *in vitro *kinase assays were carried out with bacterially expressed proteins fused to the glutathione S-transferase (GST) protein. Wild-type GST-Pim-1, but not the corresponding kinase-deficient K67M mutant was able to phosphorylate itself, the C-terminal interacting fragment of human RUNX3 as well as the full-length murine Runx3 protein, but not the GST moiety (Figure 3A and data not shown). Pim-1 phosphorylated also several C-terminal fragments of murine Runx1 (Figure 3A). Since not all the Runx1 and Runx3 fragments overlapped with each other (Figure 3B), this suggests that there are multiple target sites for Pim-1 within the RUNX proteins.

### Pim-1 potentiates transcriptional activity of the RUNX1 transcription factor

**Figure 4:**
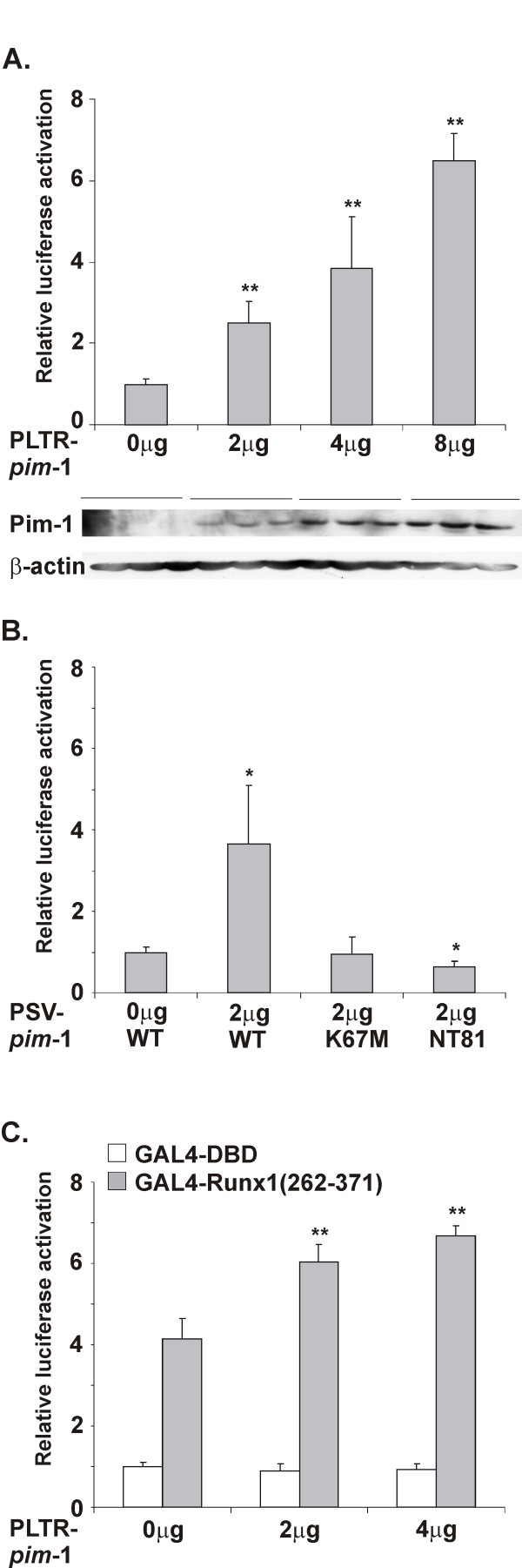
**Pim-1 potentiates transcriptional activity of Runx1**. (A) Jurkat TAg-cells were transfected with 4 μg of pM-CSF-R-Luc, 1 μg of pSV-β-gal, 4 μg of pEF-*Runx1*, 2 μg of pEF-*Cbf*β*2*, and indicated amounts of pLTR-*pim-1*. The steady-state levels of Pim-1 protein were measured from the same cell lysates by Western blotting with anti-Pim-1 antibody and equal loading was verified with anti-β-actin antibody. (B) Jurkat TAg-cells were transfected with same reporter constructs as in Figure A together with wild-type or mutant pSV-*pim*-1 constructs. (C) Jurkat TAg-cells were transfected with 3 μg of pG5-Luc, 1 μg of pSV-β-gal, 3 μg of GAL4 fusion proteins and 2 μg of pEF-*Cbf*β*2 *together with indicated amounts of pLTR-*pim*-1. Shown are relative luciferase activities normalized against β-galactosidase activities and statistically analysed by Student's t-test (*, p ≤ 0.05; **, p ≤ 0.01).

Phosphorylation by extracellular signal-regulated kinase (ERK) has previously been shown to potentiate the transactivation ability of RUNX1 [33]. To investigate whether phosphorylation of Runx1 by Pim-1 had similar consequences, transient transactivation experiments were carried out in Jurkat T cells using a previously established luciferase reporter assay with which the functional domains of Runx1 had been determined [19]. There the luciferase gene is driven by the macrophage-colony stimulating factor receptor (M-CSF-R) promoter containing binding sites for RUNX, PU.1 and C/EBP transcription factors. In Jurkat T cells, the reporter is inactive in the absence of ectopic expression of any RUNX family member and their heterodimeric binding partner CBFβ, while Runx1 alone only slightly activates it. Also in our assays, ectopic expression of Pim-1 was unable to stimulate luciferase activity in the absence of Runx1 (data not shown). As shown in Figure 4A, increasing amounts of wild-type Pim-1 were able to enhance Runx1/Cbfβ-dependent transactivation of the luciferase reporter in a statistically significant and dose-dependent fashion, as also confirmed by analysis of the steady-state levels of Pim-1 protein by Western blotting. By contrast, the kinase-deficient K67M mutant of Pim-1 did not have any major effects on Runx1 activity, while the more extensive NT81 mutant lacking the N-terminal 80 amino acids of Pim-1 even slightly inhibited it (Figure 4B). We have previously shown that this mutant can act in a dominant negative fashion to downregulate the effects of the endogenously expressed wild-type Pim-1 protein e.g. on NFATc activity [8].

To examine whether the effects of Pim-1 were mediated via the activation domain of Runx1 that Pim-1 was able to phosphorylate, additional assays were carried out with a GAL4-dependent luciferase reporter coexpressed with a fusion protein where the yeast GAL4 DNA-binding domain had been fused with the activation domain of Runx1 (amino acids 262–371) containing two major transactivation elements, TE1 and TE2 [[19], see Figure 3B]. Indeed, wild-type Pim-1 was able to increase luciferase activity when coexpressed with the GAL4-Runx1 fusion protein, but not with the GAL4 DNA-binding domain alone (Figure 4C). Since the kinase-deficient mutants remained inactive in this assay (data not shown), our results suggest that the effects of Pim-1 are dependent on the presence of its phosphorylation target sites within the activation domain of RUNX proteins.

Ser^249 ^and Ser^266 ^of RUNX1 have been shown to be targeted by the extracellular signal-regulated kinase (ERK) [33]. More recent studies have indicated that phosphorylation by ERK affects not only activity, but also localization and stability of RUNX1 [34]. Unphosphorylated RUNX1 interacts with the transcriptional repressor mSin3A and is associated with nuclear matrix. Phosphorylation of RUNX1 by the ERK-dependent pathway releases RUNX1 from mSin3A and nuclear matrix, and this is accompanied with enhanced transcriptional activity. However, since binding to mSin3A protects RUNX1 from proteosome-mediated degradation, corepressor release from RUNX1 may regulate its transcriptional activity in a time-dependent fashion, and thereby prevent prolonged RUNX1 activation in response to cytokines or growth factors. Since none of the amino acid sequences surrounding ERK-phosphorylated or other C-terminal serine or threonine residues in RUNX1 show obvious homology to the reported Lys/Arg-rich Pim-1 consensus phosphorylation site [35], the Pim-1 target sites as well as the putative effects of the Pim-1 kinase on stability of RUNX proteins remain to be identified.

### Pim-1 may regulate hematopoietic cell fate together with RUNX proteins

Enforced expression of *Runx2 *and *gfi*-1 transcription factors in murine thymocytes has been shown to result in delayed thymocyte development at the stage of β-selection where cells rearrange their T cell receptor β (TCRβ) locus [36,37]. Interestingly, Pim-1 is able to promote maturation of double negative (DN) thymocytes into double positive (DP) thymocytes in Rag-deficient and TCRβ enhancer-deleted mice, which are deficient in β-selection as are also mice overexpressing Gfi-1 [37-39]. In addition, intact Runx1 protein is required for cell proliferation during DN-to-DP transition [40]. Thus, strict spatio-temporal expression of all these proteins is important for development of DN thymocytes into more mature DP T cells and further into functional mature single positive effector T cells.

Both *pim *and *Runx *family genes can cooperate with *myc *family genes in tumor formation [1,25], which correlates well with the observation that both *Runx1 *and *Runx3 *were found among the genes that could substitute for *pim*-1 and *pim*-2 in retroviral tagging experiments [41]. However, *pim*-1 and *Runx2 *have also been shown to cooperate with each other [3], suggesting that these genes are not completely redundant in their oncogenic effects and that RUNX family transcription factors may function both in parallel as well as downstream of Pim kinase-modulated pathways.

## Conclusion

Our data indicate that the Pim-1 serine/threonine kinase is able to physically interact with the RUNX family transcription factors, colocalize with them within nuclei and phosphorylate them *in vitro*. Moreover, the transcriptional activity of at least Runx1, but most likely also of other RUNX family members is potentiated by Pim-1. These results have revealed a previously unrecognized signaling cascade involving Pim-1 kinase and the RUNX family of transcription factors that may control differentiation and transformation of hematopoietic cells.

## Methods

### Plasmids

Eukaryotic pLTR-*pim*-1, pSV-*pim*-1, pECFP-*pim*-1 and prokaryotic GST-Pim-1 fusion vectors expressing the wild-type murine protein or the kinase-deficient K67M or NT81 mutants have been described previously [8,30] as also all yeast vectors used [9], pEF-BOS, pEF-*Runx1*, pEF-*Runx3*, pEF-*Cbf*β*2 *and GAL4-*Runx1 *fusion constructs and the prokaryotic GST fusion vectors expressing murine Runx proteins and their deletion derivatives [19,24]. Two additional deletion derivatives Runx1(179–292) and Runx1(179–320) were made by digesting GST-Runx1(179–343) vector with Sal I or Sph I restriction enzymes, respectively. GST-B19 was prepared from the yeast VP16-B19 fusion vector expressing amino acids 264–404 of human RUNX3. MYC-tagged *Runx1 *and -*Runx3 *as well as FLAG-tagged *Runx1 *expression vectors were prepared by PCR from pEF-*Runx1 *and pEF-*Runx3 *plasmids and cloned into pAMC [42], kindly provided by Dr. Tomi Mäkelä, (Biomedicum Helsinki, Finland), or pFLAG-CMV-2 (Kodak) vectors, respectively. Runx proteins fused to the enhanced yellow fluorescent protein were subcloned into the pEYFP-C1 vector from Clontech. M-CSF-R-Luc reporter plasmid was kindly provided by Dr. Dong Er-Zhang (Harvard Medical School, Boston, MA), while GAL4-luciferase (G5-Luc) and pSV-β-galactosidase (pSV-β-gal) reporter plasmids were from Promega.

### Cell culture

Jurkat T cell derivatives, JTAg cells expressing the SV40 T-antigen [43] were maintained in Roswell Park Medical Institute (RPMI) medium (Sigma-Aldrich) supplemented with 10 % fetal bovine serum, 100 μg/ml streptomycin, and 100 units/ml penicillin, while Dulbecco's modified Eagle's medium (DMEM) (Sigma-Aldrich) with equal supplements was used to grow COS-7 cells.

### Yeast two-hybrid assays

Yeast two-hybrid assays were carried out essentially as previously described [9,28]. Briefly, the K67M mutant of Pim-1 fused with the LexA DNA-binding domain was used as a bait to screen a library kindly provided by Stephen Elledge (Baylor College of Medicine, Houston, Texas). The library contained cDNA clones isolated from Epstein-Barr virus-transformed human peripheral blood lymphocytes and fused to the VP16 activation domain. The yeast transformants expressing Pim-1-interacting protein fragments were double-selected for their abilities to grow on histidine-deficient plates containing 25 mM 3-aminotriazole and to produce β-galactosidase. To further verify double-positive interactions, mating assays were carried out using a modified yeast two-hybrid assay [29] with baits fused with the GAL4 DNA-binding domain. Nucleotide sequences for the positive clones were determined using an Applied Biosystems automated sequencing apparatus.

### Protein interaction assays

GST pull-down assays were carried out as previously described [30] with bacterially produced GST-fusion proteins and *in vitro *translated ^35^S-labeled Pim-1 protein. For coprecipitation assays, COS-7 cells were transfected by electroporation (220 V, 975 μF) with Gene Pulser II (Bio-Rad). Two days later, cells were collected and lysed by one freeze-thaw cycle into co-IP buffer (50 mM Tris-HCl, pH 7.5, 150 mM NaCl, 0.1 mM EDTA, 0.5 % NP-40, 20% glycerol and 1:100 Protease Inhibitor mix (Sigma-Aldrich)). 100 μg aliquots of protein were used to confirm protein expression by Western blotting, whereas 500 μg aliquots of protein were subjected to immunoprecipitation with mouse monoclonal anti-MYC (Sigma-Aldrich) or M2 anti-FLAG (Kodak) antibodies bound to protein G-sepharose beads (Amersham Biosciences) for 2 hours or overnight at 4°C. Precipitated proteins were washed 5–6 times with co-IP buffer, resolved on SDS-PAGE and transfered onto PVDF membrane (Amersham Pharmacia Biotech). To detect proteins by Western blotting, membranes were incubated with mouse monoclonal anti-Pim-1 (19F7; Santa Cruz Biotechnology), anti-MYC (Sigma-Aldrich), M2 anti-FLAG (Kodak) or anti-β-actin (Sigma-Aldrich) antibodies followed by HRP-linked anti-mouse antibodies (Zymed), and the ECL+plus chemiluminescence reagents (Amersham Biosciences).

### Cell imaging assays

COS-7 cells were transiently transfected with ECFP or EYFP fusion vectors as described above and plated on coverslips. Two days later, cells were fixed with 4% paraformaldehyde, after which confocal images were captured with Zeiss LSM510 META confocal microscope. ECFP and EYFP fusion proteins were excited with 405 nm and 514 nm laser lines and emissions were collected with BP 435–485 and LP 560 filters, respectively. The optical thicknesses of the two channels were equalized prior to image acquisition and colocalization was visualized with a scattergram plot acquired with Zeiss LSM510 3.2 program.

### In vitro kinase assays

*In vitro *kinase assays were carried out as previously described [8]. Briefly, bacterially produced GST-fusion proteins were mixed in kinase buffer (20 mM Pipes, pH 7.0, 5 mM MnCl_2_, 7 mM β-mercaptoethanol, 0.25 mM β-glycerophosphate, 0.4 mM spermine, 10 μM rATP, 1:200 aprotinin (Sigma-Aldrich) supplemented with 10 μCi of γ-^32^P-ATP (Amersham Biosciences) and incubated at 30°C for 30 minutes. Samples were separated on SDS-PAGE and visualized by autoradiography.

### Transactivation assays

5 × or 10 × 10^6 ^Jurkat TAg-cells were transfected by electroporation (250 V, 975 μF). Two days later, cells were collected and analysed for luciferase activity using Luminoskan Luminometer (Labsystems). The transfection efficiencies were normalized against β-galactosidase activities. Shown in the figures are means and standard deviations of representative examples of at least 3 independent experiments with triplicate or quadruple samples.

## Abbreviations

β-gal, β-galactosidase; DP, double positive; DN, double negative; ECFP, enhanced cyan fluorescent protein; EYFP, enhanced yellow fluorescent protein; ERK, extracellular signal-regulated kinase; GST, Glutathione S-transferase; HRP, horse-radish peroxidase; Luc, luciferase; M-CSF-R, macrophage-colony stimulating factor receptor; PVDF, polyvinylidene fluoride; TCR, T cell receptor

## Authors' contributions

TLTA carried out protein interaction assays, transactivation assays and most of *in vitro *kinase assays, and drafted the manuscript. JS performed imaging assays and KJP yeast mating assays. YI provided reagents and expertise on Runx proteins and critically revised the manuscript. PJK initiated the project by carrying out the yeast two-hybrid screen and some *in vitro *kinase assays, and supervised the work as well as writing of the manuscript. All authors read and approved the final manuscript.
